# Tannins and Bacitracin Differentially Modulate Gut Microbiota of Broiler Chickens

**DOI:** 10.1155/2018/1879168

**Published:** 2018-02-21

**Authors:** Juan María Díaz Carrasco, Enzo Alejandro Redondo, Natalia Daniela Pin Viso, Leandro Martin Redondo, Marisa Diana Farber, Mariano Enrique Fernández Miyakawa

**Affiliations:** ^1^Instituto de Patobiología, Centro Nacional de Investigaciones Agropecuarias, Instituto Nacional de Tecnología Agropecuaria, Calle Las Cabañas y Los Reseros s/n, Casilla de Correo 25, Castelar, 1712 Buenos Aires, Argentina; ^2^Consejo Nacional de Investigaciones Científicas y Técnicas, Godoy Cruz 2290, 1425 Ciudad Autónoma de Buenos Aires, Argentina; ^3^Instituto de Biotecnología, Centro Nacional de Investigaciones Agropecuarias, Instituto Nacional de Tecnología Agropecuaria, Calle Las Cabañas y Los Reseros s/n, Casilla de Correo 25, Castelar, 1712 Buenos Aires, Argentina

## Abstract

Antibiotic growth promoters have been used for decades in poultry farming as a tool to maintain bird health and improve growth performance. Global concern about the recurrent emergence and spreading of antimicrobial resistance is challenging the livestock producers to search for alternatives to feed added antibiotics. The use of phytogenic compounds appears as a feasible option due to their ability to emulate the bioactive properties of antibiotics. However, detailed description about the effects of in-feed antibiotics and alternative natural products on chicken intestinal microbiota is lacking. High-throughput sequencing of 16S rRNA gene was used to study composition of cecal microbiota in broiler chickens supplemented with either bacitracin or a blend of chestnut and quebracho tannins over a 30-day grow-out period. Both tannins and bacitracin had a significant impact on diversity of cecal microbiota. Bacitracin consistently decreased* Bifidobacterium* while other bacterial groups were affected only at certain times. Tannins-fed chickens showed a drastic decrease in genus* Bacteroides* while certain members of order Clostridiales mainly belonging to the families Ruminococcaceae and Lachnospiraceae were increased. Different members of these groups have been associated with an improvement of intestinal health and feed efficiency in poultry, suggesting that these bacteria could be associated with productive performance of birds.

## 1. Introduction

For more than 50 years, antibiotic growth promoters (AGPs) have been used in agricultural animal production as a means to increase growth performance through maintained animal health and improved feed efficiency [1]. During the last decades, global concern about development and transference of antimicrobial resistance from animal to human strains is rising [2]. The benefits of AGPs use in production animals are often argued to be outweighed by their negative effects and this practice has been discontinued in the European Union since 2006 due to increasing concern over the spread of antibiotic resistance genes to human pathogens [3]. On the other hand, an important and growing consumer demand for antibiotic-free poultry products is pressing to use cost effective alternatives to AGPs [4, 5].

Although it is still unclear how AGPs enhance animal performance, it is speculated that they act mainly through modulation of gastrointestinal microbiota [6, 7]. The chicken intestinal microbiota plays an important role in digestion and conversion of food into body mass [8, 9] and also in protection from pathogens, detoxification, and modulation of the immune system [10, 11]. Many studies on poultry microbiota have used the cecum as sampling site due to its relationship with chicken productivity and the highly diverse bacterial communities that inhabit this section of the intestine. The cecum is an important organ contributing to intestinal health and nutrition of birds where anaerobic fermentation of cellulose, starch, and other resistant polysaccharides is performed [12, 13].

Much research has been done in order to characterize the intestinal microbiota of poultry. Initially, most of these works have relied on culture-dependent approaches [14]; and more recently, culture-independent methods have been employed such as denaturing gradient gel electrophoresis, restriction fragment length polymorphisms, and clone libraries, in an effort to overcome the limitations and biases associated with culture-based techniques, since a large portion of the microorganisms comprising the microbiota are not cultivable [15–17]. The advent of high-throughput sequencing of 16S rRNA gene amplicons has enabled the study of bacterial communities at increased depth and resolution [18]. This technology has been used to describe the functional diversity [19] and natural variability of cecal microbiota [20, 21], as well as the temporal [22, 23] and spatial [24–26] variations that normally exist in the chicken gastrointestinal microbiota.

Bacitracin is a mixture of high molecular weight polypeptides that possess antimicrobial activity against gram-positive microorganisms interfering with formation of the bacterial cell wall [27]. Bacitracin is one of the most extensively used AGPs to improve productivity in poultry [1]. In calves, bacitracin has been shown to alter fecal microbiota composition but did not improve animal performance [28]. Some studies have reported alterations in the gut bacterial community of broiler chickens associated with dietary supplementation with bacitracin [17, 29].

Among the available alternatives to replace AGPs for poultry industry, phytogenic additives appear as candidates due to their ability to emulate the bioactive properties of conventional AGPs [30]. Tannins are polyphenolic compounds widely distributed in the plant kingdom, where they play a protective role [31]. Tannins added to the diet are being used in farm animals to improve nutrition and control enteric diseases [32, 33]. However, the effects of tannins on the chicken gut microbiota remain unclear since previous studies have often relied on* in vitro* observations or culture-dependent methods which fail to provide an accurate description of the taxonomic composition and bacterial community structure of chicken microbiota. The aim of the present study was to comparatively analyze the differential effects of dietary supplementation with tannins and bacitracin on chicken cecal microbiome by means of high-throughput sequencing of 16S rRNA gene amplicons.

## 2. Materials and Methods

### 2.1. Chicken Diets and Experimental Design

A total of 120 one-day-old unvaccinated male Cobb chicks were obtained from a local commercial hatchery and grown over a 30-day period in biosafety level 2 facilities located at Veterinary and Agriculture Research Center (CICVyA-INTA). Studies presented here were reviewed and approved by the CICVyA-INTA Institutional Animal Care and Use Committee under protocol number 20/2010.

Birds were randomly divided into three groups (40 chicks per group) corresponding to the following dietary treatments: (1) CON: control diet without any supplements; (2) BAC: diet supplemented with subtherapeutic levels of zinc bacitracin (1 g/kg of feed); (3) TAN: diet supplemented with a blend of tannins derived from chestnut* (Castanea sativa)* and quebracho* (Schinopsis lorentzii)* (1 g/kg of feed). Dietary treatments were prepared by thoroughly mixing commercial starter feed (3200 kcal/kg; 20% protein; Alimcer S.A., Buenos Aires, Argentina) with the corresponding supplements. Chickens had ad libitum access to feed and water. Each experimental group was housed in a floor pen (1.5 × 1.5 × 0.8 m) made of 0.55 mm wire mesh and hardboard pieces covering the lower part of the mesh, each containing a galvanized steel self-feeder and a waterer. Birds were raised under controlled environmental conditions and automated ventilation system with 18-hour lighting cycle and a temperature of 32°C on day 1, which was gradually diminished and maintained to 24°C on day 15. Prior to chick placement on pens, litter from a previous flock in which no supplements were used was thoroughly mixed with fresh commercial wood shavings and placed into all pens. On day 21, each group of birds was randomly split in two pens in order to avoid overcrowding and maintain animal density. Body weight (BW) of each animal and feed consumed by each treatment group were recorded on days 5, 12, 19, 26, and 30. Feed conversion ratio (FCR) was calculated as the ratio of feed intake (kg) and weight gained (kg) for each group.

### 2.2. Sample Collection and DNA Extraction

On days 12, 19, 26, and 30, five animals per group were euthanized by cervical dislocation and both cecal lobes were removed from each bird. The samplings were always carried out at 10 AM, six hours after the start of the light phase of the photoperiod. The tips of the cecal lobes were cut off, and cecal contents were aseptically squeezed out and pooled into sterile recipients for each group. Samples were immediately refrigerated on ice and stored at −80°C until DNA extraction. Total DNA was isolated from 300 mg of cecal contents using QIAamp DNA Stool Mini Kit (Qiagen, Hilden, Germany) following manufacturer instructions. DNA concentration and quality were assessed in NanoDrop ND-1000 spectrophotometer (NanoDrop Technologies, DE, USA). DNA was stored at −20°C until further analysis.

### 2.3. 16S rRNA Gene Library Preparation and High-Throughput Sequencing

The 16S rRNA gene V3-V4 regions were amplified using Illumina primers (forward 5′CCTACGGGNGGCWGCAG 3′, reverse 5′GGACTACHVGGGTATCTAATCC 3′) with standard adapter sequences attached for barcoding and multiplexing. 16S rRNA gene libraries construction and high-throughput sequencing were performed at Macrogen Inc. (Seoul, South Korea) using the Illumina MiSeq platform following manufacturer's instructions for 2 × 300 bp paired-end sequencing protocol [34]. In order to reduce unbalanced and biased base compositions, 15% of PhiX control library was spiked into the amplicon pool. The datasets generated in this study are available under request.

### 2.4. Sequence Preprocessing

Primer and adapter sequences were trimmed using Trimmomatic v0.33 [35], also removing leading and trailing bases. Paired-end reads were merged into single contigs with FLASh v1.2.11 [36]. Reads were demultiplexed and filtered using a threshold Phred quality score of *Q* > 20. Chimeric sequences were filtered out using USERCH algorithm [37].

### 2.5. Microbial Community Analysis

Microbial composition and diversity were analyzed using Quantitative Insights into Microbial Ecology (QIIME) software v1.9.1 [38] with default parameters, unless specified. Open-reference operational taxonomic units (OTUs) picking was performed using UCLUST and USEARCH algorithms. Taxonomy was assigned against the Greengenes reference OTU build version 13.8, using a 97% sequence similarity threshold. OTUs with abundance below 0.005% were filtered out from the final OTU table. Normalization of OTU counts was done by performing multiple rarefactions with steps of 1.000 reads and 100 iterations at each rarefaction depth. Alpha diversity was calculated through richness (number of OTUs) and diversity (Shannon's index) estimators. Principal coordinate analysis (PCoA) plots were generated in QIIME based on unweighted UniFrac distance matrix. This method is a *β*-diversity measure that takes into account the phylogenic divergence between OTUs to identify differences in the overall microbial community structure between samples [39].

### 2.6. Statistical Analysis

The relative abundances of bacterial populations were analyzed using Statistical Analysis of Metagenomic Profiles (STAMP) software [40]. Relative abundances were compared by two-tailed Fisher's exact test with Storey's FDR correction at each level of classification (phylum, class, order, family, and genus). Additionally, when comparing pairs of cecal samples, STAMP was set to only consider taxa represented by at least 50 sequences and an effect size filter of 3.00. Comparisons on growth performance parameters and diversity estimators between groups of samples were calculated using nonparametric Kruskal−Wallis test and two-tailed Mann−Whitney test for pairs of groups (GraphPad Software, CA, USA), which were considered statistically significant if *p* < 0.05. Calculation of unweighted UniFrac *β*-diversity metric was subjected to nonparametric permutational analysis of variance (PERMANOVA) in QIIME with 1.000 permutations in order to assess significant differences between samples taken at different time points and between dietary treatments.

## 3. Results

A total of 1.129.286 paired-end reads were obtained from 12 cecal samples. After quality filtering and removal of chimeric reads, 619.152 sequences remained covering complete V3-V4 regions of the 16S rRNA gene, with a mean length of 452 ± 10 bp. The average number of reads per cecal sample was 51.596 ± 12.406 bp. A total of 513 operational taxonomic units (OTUs) with abundance greater than 0.005% were obtained from all samples.

### 3.1. Impact of Dietary Treatments on Diversity of Cecal Microbiota

**Figure 1:**
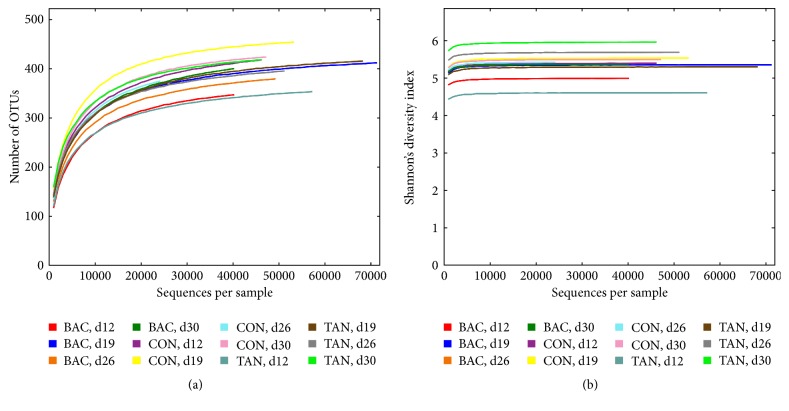
Rarefaction curves of (a) number of OTUs and (b) Shannon's index, obtained based on 16S rRNA gene V3-V4 sequences. OTUs were picked using the UCLUST method with 3% dissimilarity in QIIME. Each curve corresponds to a single pooled cecal sample.

**Figure 2:**
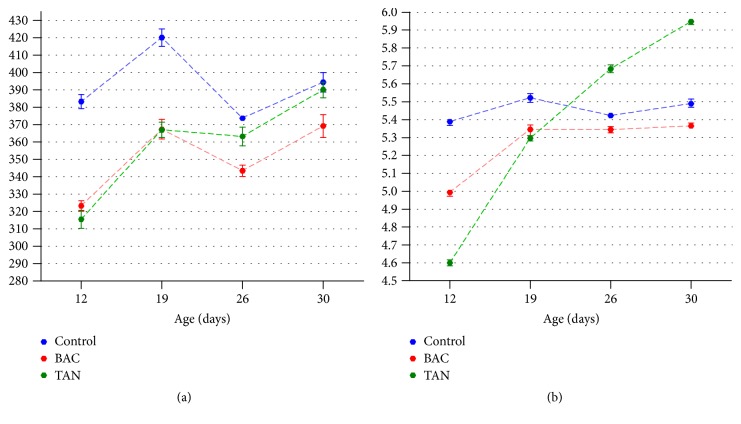
Effect of tannins and bacitracin supplementation on (a) the number of OTUs and (b) Shannon's diversity index of cecal microbiota over time. Bars indicate SD.

Internal sample *α*-diversity was estimated through the number of OTUs (richness) and Shannon's index (diversity). Rarefaction curves of observed OTUs (Figure 1(a)) and Shannon's index values (Figure 1(b)) reached a plateau in all samples, demonstrating that sequencing depth was adequate to cover the bacterial diversity in poultry cecal samples. The overall average number of OTUs per sample was 368 ± 29, while average Shannon's index was 5.37 ± 0.32. Alpha diversity estimators varied significantly with both treatments as well as with the age of sampling (*p* < 0.001). Between days 12 and 26, animals treated with tannins and bacitracin showed significantly lower richness than the control group (Figure 2(a)). At day 30, tannins-supplemented birds reached a number of OTUs similar to that of the control, while cecal richness of bacitracin-treated animals remained significantly lower. Shannon's diversity index showed a similar profile, although more complex bacterial communities were evidenced in tannins-supplemented birds between days 26 and 30 (Figure 2(b)).

**Figure 3:**
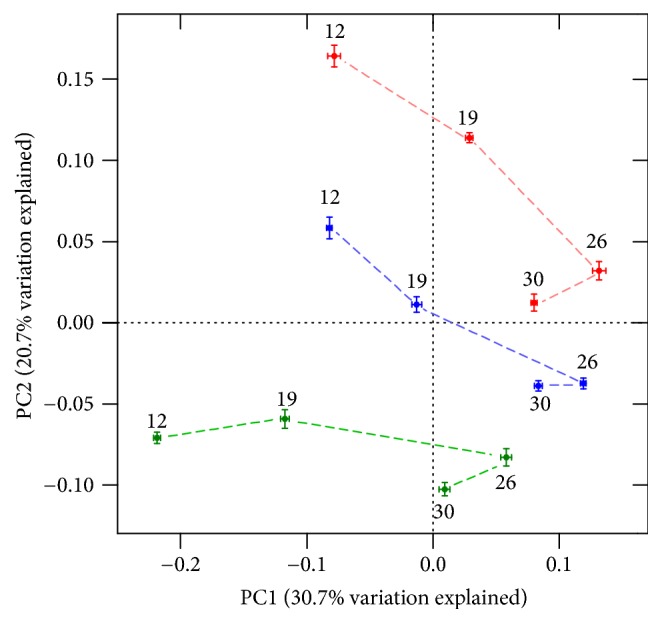
PCoA plot based on unweighted UniFrac metric. Each color represents a different dietary treatment (blue: control without additives; red: bacitracin; green: tannins). Numbers by each point indicate the age of sampling in days. Axes (PC1 = 30.7% and PC2 = 20.7%) account for 51.4% of total variation observed. Bars indicate SD.

A principal coordinate analysis (PCoA) based on unweighted UniFrac distances was conducted to determine any separation into sample clusters (Figure 3). PCoA plots revealed that the samples corresponding to each dietary treatment form separate series, indicating that tannins and bacitracin differentially modulate cecal microbiota. The PERMANOVA analysis detected significant changes on *β*-diversity among dietary treatments (*p* = 0.031) and among sampling times (*p* = 0.019), which is consistent with the evident temporal structure of the data depicted in the PCoA plot.

### 3.2. Effects of Tannins and Bacitracin on Composition of Cecal Microbiota

At the phylum level, cecal microbiota was dominated by Firmicutes (CON: 49.29%, BAC: 46.28%, and TAN: 54.00%) and Bacteroidetes (CON: 45.03%, BAC: 48.57%, and TAN: 39.97%), followed by Proteobacteria (CON: 3.90%, BAC: 3.65%, and TAN: 3.16%) and Actinobacteria (CON: 1.58%, BAC: 1.09%, and TAN: 2.39%). The other two phyla, Deferribacteres (CON: 0.10%, BAC: 0.29%, and TAN: 0.37%) and Tenericutes (CON: 0.02%, BAC: not detected, and TAN: 0.03%), were detected in specific samples. Less than 0.10% of the sequences remained unclassified.

**Figure 4:**
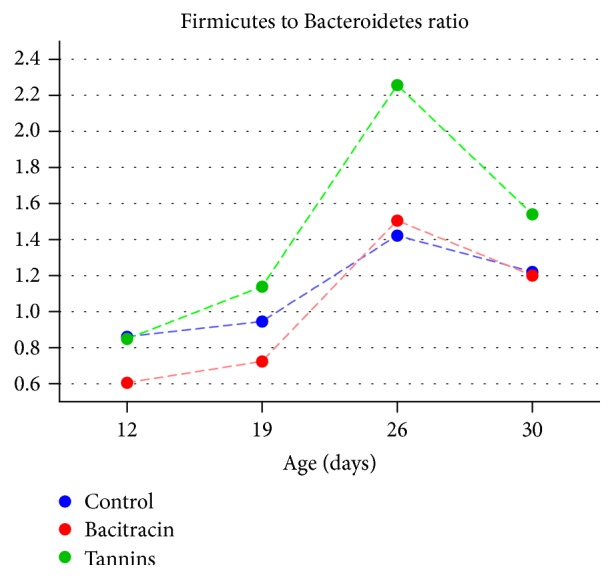
FBR of CON, BAC, and TAN treated chickens over time.

The abundances of the two predominant phyla, Firmicutes and Bacteroidetes, showed a strong inverse correlation (Spearman *R* = −0.958, *p* < 0.0001). The Firmicutes to Bacteroidetes ratio (FBR) showed variations over time and across treatments (Figure 4). At day 12, Firmicutes were significantly more abundant in the CON and TAN groups (42.51% and 42.66%, resp.) than in BAC treated chicks (34.89%), while Bacteroidetes showed the opposite pattern. At day 19, BAC treated animals had a lower proportion of Firmicutes than the CON group (38.99% and 45.09%, resp.). At days 26 and 30, no significant differences in the abundance of Firmicutes or Bacteroidetes between the CON and BAC groups were detected. From days 19 to 30, the TAN group exhibited a significantly higher abundance of Firmicutes than CON and BAC treatments.

**Figure 5:**
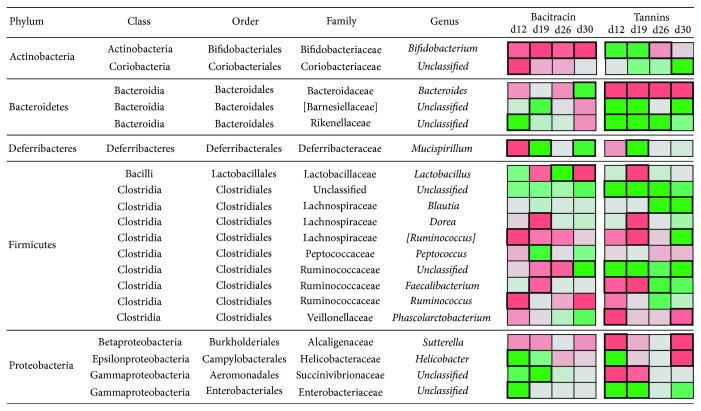
Effects of tannins and bacitracin in the relative abundance of different bacterial groups of cecal microbiota over time. The heatmap in the right depicts the changes in the relative abundance of each of the taxa with respect to that observed in the control group (green: increased abundance; red: decreased abundance). Cells boxed with thick lines indicate statistically significant changes detected with STAMP.

Different bacteria of the cecal microbiota were affected by tannins and bacitracin, and the impact of each treatment also varied depending on the age of sampling. Twenty bacterial taxa were significantly altered by the treatment with BAC or TAN at least at one time point (Figure 5 and Supplementary Figure 1).

*Bacteroides* was the most abundant genus on average (21.90%), but these bacterial taxa showed a drastic decrease in the TAN group at all the time points analyzed. Other less abundant bacterial species were significantly affected by the TAN treatment at day 12, including the genera* Phascolarctobacterium*,* Sutterella*, and* Faecalibacterium* and unclassified members of family Succinivibrionaceae. The decline of genus* Bacteroides* in TAN treated chicks was compensated by an increase of other Bacteroidetes belonging to the families Rikenellaceae and Barnesiellaceae and also by the increase of the Firmicutes, including members of order Clostridiales and family Ruminococcaceae (at all the times analyzed), and genus* Blautia *(at days 26 and 30). An increase was also observed in bacteria belonging to phylum Actinobacteria (*Bifidobacterium* at days 12 and 19 and members of the family Coriobacteriaceae between days 19 and 30) and phylum Proteobacteria (members of the family Enterobacteriaceae at days 12 and 19).

Chickens supplemented with bacitracin showed a different cecal microbiota profile than those treated with tannins. Bacitracin did not significantly affect genus* Bacteroides* but increased them by 4% at day 30 with respect to the control group. At day 12, BAC treatment impacted on a group of bacteria that included genera* Mucispirillum*,* [Ruminococcus]*,* Ruminococcus*, and* Bifidobacterium* and an unclassified member of the family Coriobacteriaceae. The negative effect of bacitracin on Actinobacteria was repeatedly observed at subsequent sampling times, particularly for the genus* Bifidobacterium*. Genera* Ruminococcus* and* [Ruminococcus]* were also lowered by bacitracin between days 19 and 30. On the other hand, bacitracin favored different taxa at each age of sampling. In the first two sampling times, BAC treatment significantly enhanced members of phyla Proteobacteria (genus* Helicobacter* and families Enterobacteriaceae and Succinivibrionaceae) and Bacteroidetes belonging to the families Rikenellaceae and [Barnesiellaceae], as well as genera* Mucispirillum* and* Peptococcus*. At day 26, a strong increase of genus* Lactobacillus* was detected in BAC treated chicks (CON: 2.17%; BAC: 8.39%), although the opposite pattern was observed at day 30 (CON: 5.36%; BAC: 1.63%). Three taxa from different phyla were significantly enhanced in BAC treated animals at day 30:* Bacteroides*,* Mucispirillum*, and an unclassified member of family Ruminococcaceae.

### 3.3. Growth Performance

Results for production traits of broilers through the experimental period are shown in Table 1. The average body weight of chickens did not differ significantly among treatments throughout the breeding cycle.

## 4. Discussion

Detailed description about the effects of classic AGPs and alternative phytogenic compounds on chicken intestinal microbiota is necessary to understand the underlying mechanisms of growth promotion. Improvements in feed conversion associated with dietary supplementation with antibiotics are thought to involve gastrointestinal microbial communities, but this connection remains poorly understood. The establishment of an adult microbiota is a complex process that is influenced by numerous factors including host genetics, intestinal health, stress, age, breeding conditions, weather conditions, diet, litter composition, and the use of feed additives [9, 18, 41, 42]. In the present study, high-throughput sequencing of 16S rRNA gene was used to monitor bacterial composition of the cecal microbiota in chickens supplemented with either bacitracin or tannins over a 4-week production cycle. We found that tannins and bacitracin have a distinct impact on cecal microbiota, each one affecting different bacterial groups at each sampling time.

Analyses of rarefaction curves and diversity indexes indicate that microbial richness and diversity significantly changed with age and dietary treatments. Previous reports found that bacterial diversity in the cecum increases with the age of the bird [18, 23, 43, 44]. This observation was corroborated by our data, since Shannon's diversity index was higher at day 30 than at day 12 in all groups, but the increase was more pronounced in tannins treated birds. There is evidence suggesting that higher diversity microbiota is beneficial in chickens but the cause and effect relationships have not been elucidated [8]. On the other hand, bacitracin-treated chicks showed lower diversity parameters than the control at all the time points analyzed. Lu et al. (2008) also detected a reduction in the gastrointestinal microbiota diversity when bacitracin was administered [29]. However, other authors found that the overall microbial diversity is not significantly disturbed by bacitracin and other AGPs [43, 45–48], although changes in the relative abundance of certain taxa were described in each case.

Although much research has been done regarding the effects of AGPs on the intestinal microbiota of poultry, detailed information about the impact of phytogenic feed additives on chicken microbiota is still lacking. Different tannins including those derived from chestnut and quebracho have shown activity against* Clostridium perfringens* and other poultry pathogens both* in vitro* and* in vivo* [30]. Chickens fed tannin-rich grape products showed increased diversity in the cecum, and these effects correlated with the detection of several potential tannins-degrading bacteria and higher counts of* Lactobacillus* and* Enterococcus* at 21 days of age [49]. Our results indicate that inclusion of tannins in the diet increased cecal diversity between days 26 and 30, although this effect was not associated with the abundance of members of class Bacilli but with bacteria belonging mainly to families Ruminococcaceae and Lachnospiraceae.

Previous studies showed that dietary supplementation with AGPs alters the composition of chicken microbiota, mainly affecting lactobacilli and other Firmicutes in the proximal section of the gastrointestinal tract [50, 51]. A recent work showed that virginiamycin supplementation increased the relative abundance of genus* Propionibacterium* in the ileum, which correlated with a higher concentration of propionate in the cecum, and the authors hypothesized that these bacteria may contribute to the reported growth promoting effects of AGPs [45]. Other authors found significant changes in the cecal microbiota composition of chickens treated with monensin in the presence of AGPs tylosin and virginiamycin, which reduced lactobacilli and enterococci and modulated the abundance of members of the families Ruminococcaceae and Lachnospiraceae [44]. We found that bacitracin impacted on several members of the families Ruminococcaceae and Lachnospiraceae mainly represented by genera* Ruminococcus* and* [Ruminococcus]*, respectively, while the enrichment in an unclassified Ruminococcaceae was detected at day 30. On the other hand, tannins treatment consistently and strongly increased unclassified members of order Clostridiales and family Ruminococcaceae, as well as the levels of other classified genera of the families Ruminococcaceae and Lachnospiraceae at 26 and 30 days of age. Previous reports have found a cecal enrichment in different member of order Clostridiales after dietary supplementation with AGPs, including unclassified Clostridiales and members of the families Ruminococcaceae and Lachnospiraceae [46, 50, 52]. Moreover, some authors have suggested that these microorganisms could be developed as poultry probiotics [17, 47, 53].

The ratio between phyla Firmicutes and Bacteroidetes in the gut microbiota has been linked to the efficiency in energy harvesting in different animals including mice, pigs, cows, and humans [54–57], suggesting a correlation between growth performance and FBR. In all the analyzed samples, regardless of the dietary treatment, cecal microbiota was dominated by phyla Firmicutes and Bacteroidetes, comprising 94% of the sequences, which is in line with previous reports [12, 13, 52, 58, 59]. A significant increase in the FBR in tannins treated animals was observed in comparison with CON and BAC groups, but this parameter showed no correlation with BW in any of the treatments. Stanley et al. (2013) showed that FBR in the cecum is variable among individual chickens from the same flock but this parameter was not correlated with growth performance [58]. Similarly, other authors found no significant correlation between FBR in the cecum and BW [52, 60]. However, other studies have found a concomitant increase in FBR and FCR in both cecal [52] and fecal [61] microbiota of chicken. The lack of a significant performance response with BAC and TAN supplementation in this study is not surprising given the small number of birds employed and the highly sanitized experimental conditions used, which may not faithfully reproduce the productive conditions.

Interestingly, the higher abundance of members of order Clostridiales has been linked to improved performance of chicken when analyzing both cecal [53, 62, 63] and fecal microbiota [61]. Further research about the specific taxa of order Clostridiales that are associated with growth promotion is required in order to identify and develop new probiotics and prebiotics for poultry.

The presence of probiotic bacteria in the intestine of chicken is associated with an improvement in the performance parameters and a reduction of pathogen loads [8, 64, 65]. Many studies have documented a reduction in the chicken intestinal load of probiotic bacteria after administration of AGPs, including lactobacilli, bifidobacteria, and enterococci [16, 29, 43, 44, 50, 66].

Lactic acid bacteria, especially* Lactobacillus* strains, have been considered as excellent probiotic microorganisms because of their activities in reducing the enteric diseases and maintaining healthy poultry [64]. In this study, genus* Lactobacillus* showed an oscillating pattern in BAC treated chicks, with a strong increase at day 26 and a sharp fall at day 30 with respect to the control group. Our results show that the effect of bacitracin on lactobacilli populations may vary with the age of sampling, similarly to what has been described for other factors such as the analyzed section of the gut [26, 46, 51] or the rearing conditions [47, 48]. Other studies have also found that certain species of lactobacilli can be favored by AGPs [17, 46].

A clear difference was observed between the effects of bacitracin and tannins on the genus* Bifidobacterium*. Tannins increased the abundance of bifidobacteria in the first two sampling times while bacitracin lowered them throughout all the breeding cycle. Previous studies have described a reduction in the cecal counts of bifidobacteria in chickens fed bacitracin and other AGPs [16, 43, 66]. On the other hand, tannins-rich grape products have been found to favor lactobacilli and to a lesser extent bifidobacteria, and it has been suggested that tannins might act as prebiotics, stimulating the proliferation of probiotic bacteria [49]. In line with this, inclusion of mannanoligosaccharides [66] and xylooligosaccharides [45] prebiotics in the diet of chickens has been shown to increase the abundance of lactobacilli and bifidobacteria. Tannins did not affect the levels of* Lactobacillus* and* Bifidobacterium* at day 30 whereas bacitracin significantly affected both of these genera. This differential effect of tannins on probiotic bacteria could contribute to improvement of bird health and reduce pathogens burden at the end of the breeding cycle of poultry.

The breakdown of nondigestible plant carbohydrates originating from the diet of herbivores leads to the formation of fermentation short-chain fatty acids (SCFAs), mainly acetate, propionate, and butyrate. The molar ratio between SCFAs has been linked to the composition of intestinal microbiota in poultry [67]. Previous studies have found that butyrate reduces shedding of acid-sensitive pathogens such as* Salmonella* in poultry and improves the growth of epithelial cells in piglets [68, 69].

Our results show that genus* Bacteroides* was drastically reduced by tannins but not by bacitracin, and this decline was mainly compensated by the increase of Bacteroidetes from the families Rikenellaceae and Barnesiellaceae, as well as the increase of Firmicutes from the families Ruminococcaceae and Lachnospiraceae. Nonadherent* Bacteroides* species have been shown to outcompete gram-positive bacteria such as Firmicutes for easily hydrolysable starch, while the latter are specialized in the degradation of a wide variety of recalcitrant substrates and persist as part of the fibrolytic communities [70].* Bacteroides* are gram-negative saccharolytic and proteolytic microorganisms that play an important role in breaking down complex macromolecules and generate acetate and propionate as main fermentation products [71, 72]. Previous studies found that polyphenols can inhibit the growth of certain* Bacteroides* while other species within this genus are favored by polyphenols [73, 74]. Families Barnesiellaceae and Rikenellaceae belong to the order Bacteroidales which encompass gram-negative anaerobic coccobacilli, with saccharolytic and proteolytic activities. Barnesiellaceae is a proposed taxonomic group which has not been yet characterized. Rikenellaceae have been found enriched in the ceca of mice with high-fat diet-induced obesity [75] and seem to be highly susceptible to perturbations in the gut microbiota such as those caused by antibiotics or probiotics supplementation [76, 77].

On the other hand, order Clostridiales encompasses mostly nonpathogenic commensal bacteria including members that have been associated with prevention of inflammatory bowel disease and maintenance of mucosal homeostasis, which has been attributed to the capacity of clostridia to produce butyrate [78]. Moreover, high-concentration butyrate-producing clostridia were isolated from the cecal content of chickens [79]. Laying hens fed tea polyphenols showed increased cecal concentration of butyrate, which protected the duodenal cells from apoptosis [80]. Mašek et al. (2014) reported an increase in the total SCFAs concentration in chickens supplemented with tannic or gallic acids [81]. It is possible that the increase of members of the families* Lachnospiraceae* and* Ruminococcaceae* observed in tannins-fed chickens could alter the SCFAs profile in the cecum towards butyrate production.

## 5. Conclusions

Taken together, our study indicates that tannins and bacitracin have a differential impact on the composition and diversity of cecal microbiota in poultry. An increase in FBR was observed in tannins-fed chickens at the end of the breeding cycle and at the same time a cecal enrichment in bacteria belonging to the order Clostridiales was detected. The abundance of different members of order Clostridiales has been linked to an improvement in the intestinal health and energy harvesting efficiency in poultry, suggesting that these taxa could be associated with growth performance. However, the mechanisms by which tannins modulate the gut ecology are still poorly understood. Further investigation utilizing full shotgun sequencing metagenomics as well as the measurement of SCFAs concentrations in the gut of chickens will shed light on this issue.

## Figures and Tables

**Table 1:** Growth performance of broilers in different treatment groups.

Parameter	Treatments		
	CON	BAC	TAN
BW (g)			
Day 12	337 ± 28	348 ± 27	323 ± 43
Day 19	777 ± 68	821 ± 77	768 ± 106
Day 26	1444 ± 122	1481 ± 153	1452 ± 224
Day 30	1814 ± 222	1905 ± 232	1798 ± 310
FCR	1.83	1.92	1.99

## Abbreviations

AGPs: Antibiotic growth promoters
CON: Control
BAC: Bacitracin
TAN: Tannins
BW: Body weight
OTUs: Operational taxonomic units
PCoA: Principal coordinates analysis
FBR: Firmicutes to Bacteroidetes ratio
FCR: Feed conversion ratio
SCFAs: Short-chain fatty acids.

## Supplementary Materials

Supplementary Materials Supplementary Figure  1: effects of tannins and bacitracin in the relative abundance of different bacterial groups of cecal microbiota over time. Colored bars show the abundance of taxa for each treatment group (blue: control; red: bacitracin; green: tannins). For each triad of bars corresponding to a single taxonomic group at a given sampling age, different letters denote treatments with significantly different relative abundances.
