# Supplementary figures and images for: Tannins and Bacitracin Differentially Modulate Gut Microbiota of Broiler Chickens
Source: Biomed Res Int. 2018 Feb 21;2018:1879168. doi: 10.1155/2018/1879168 (PMC5841071; doi:10.1155/2018/1879168)

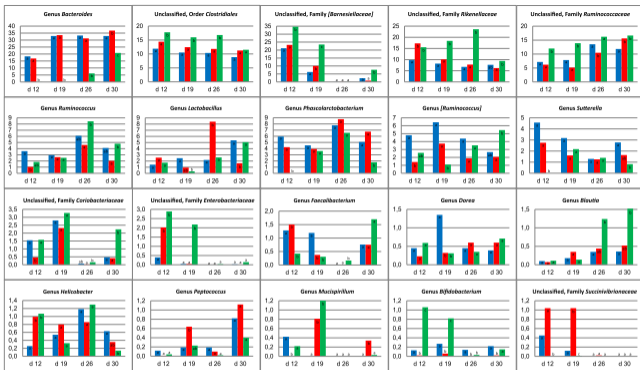

Supplement: Supplementary Materials — Supplementary Figure 1: effects of tannins and bacitracin in the relative abundance of different bacterial groups of cecal microbiota over time. Colored bars show the abundance of taxa for each treatment group (blue: control; red: bacitracin; green: tannins). For each triad of bars corresponding to a single taxonomic group at a given sampling age, different letters denote treatments with significantly different relative abundances. [file 1879168.f1.pdf]
